# Retraction: A wormhole attack detection method for tactical wireless sensor networks

**DOI:** 10.7717/peerj-cs.1449/retraction

**Published:** 2024-03-22

**Authors:** PeerJ Editorial Office

Retraction: The Publisher was alerted that prior to the submission of this article, the identical article had been submitted to other journals by authors other than Ke Zhang. We confirmed this was the case and we reached out to Ke Zhang for an explanation. We have received no reply from Ke Zhang and so we are forced to assume that this article was inappropriately copied by someone who had access to it via one of the earlier submissions. This publication is therefore Retracted and we have reported this situation to JiuZhou Polytechnic.

PeerJ Editorial Office. 2024. Retraction: A wormhole attack detection method for tactical wireless sensor networks. PeerJ Computer Science 10:e1449/retraction https://doi.org/10.7717/peerj-cs.1449/retraction

